# In Silico Exploration and Experimental Validation of *Camellia sinensis* Extract against *Rhipicephalus microplus* and *Sarcoptes scabiei*: An Integrated Approach

**DOI:** 10.3390/life13102040

**Published:** 2023-10-11

**Authors:** Mohammed Ageeli Hakami, Nosheen Malak, Afshan Khan, Hidayat Ullah, Raquel Cossío-Bayúgar, Nasreen Nasreen, Sadaf Niaz, Adil Khan, Chien-Chin Chen

**Affiliations:** 1 Department of Clinical Laboratory Sciences, College of Applied Medical Sciences, Al-Quwayiyah, Shaqra University, Riyadh 11911, Saudi Arabia; m.hakami@su.edu.sa; 2 Department of Zoology, Abdul Wali Khan University Mardan, Mardan 23200, Pakistan; 3 Centro Nacional de Investigación Disciplinaria en Salud Animal eInocuidad, INIFAP, Km 11 Carretera Federal Cuernavaca-Cuautla, No. 8534, Col. Progreso, Jiutepec 62550, Mexico; 4 Department of Zoology and Botany, Bacha Khan University, Charsadda 24420, Pakistan; 5 Department of Pathology, Ditmanson Medical Foundation Chia-Yi Christian Hospital, Chiayi 600, Taiwan; 6 Department of Cosmetic Science, Chia Nan University of Pharmacy and Science, Tainan 717, Taiwan; 7 PhD Program in Translational Medicine, Rong Hsing Research Centre for Translational Medicine, National Chung Hsing University, Taichung 402, Taiwan; 8 Department of Biotechnology and Bioindustry Sciences, College of Bioscience and Biotechnology, National Cheng Kung University, Tainan 701, Taiwan

**Keywords:** *Sarcoptes scabiei* glutathione transferase, *Rhipicephalus microplus* glutathione transferase, molecular docking, plant extract, *Camellia sinensis*

## Abstract

*Sarcoptes scabiei* is an ectoparasite of humans and animals that causes scabies. The *Rhipicephalus (Boophilus) microplus* is a blood-sucking ectoparasite that transmits various pathogens. These two parasites have caused great losses to a country’s dairy and agriculture sectors. The aim of this study was to determine the in vitro and in silico efficacy of *Camellia sinensis* plant extracts. Different concentrations of *C. sinensis* ethanolic plant extracts were prepared using the maceration method and were used against mites and ticks (in adult immersion test AIT and larval packet test LPT) to evaluate their in vitro acaricidal activity. Additionally, in silico molecular docking was performed to investigate the inhibitory interactions between the phytochemicals of the plant and *S. scabiei* and *R. microplus* glutathione transferase proteins (SsGST and RmGST). This study observed that the plant extract showed high efficacy in vitro against mites and different tick stages in adult immersion and larval packet tests. Additionally, the in silico study revealed a strong binding interaction between ellagic acid and SsGST protein, with a binding energy of −7.3 kcal/mol, with respect to permethrin (−6.7 kcal/mol), whereas quercetin and RmGST resulted in a docking score of −8.6 kcal/mol compared to deltamethrin (−8.2 kcal/mol). Overall, this study explored the potential of *C. sinensis* as a natural alternative for controlling tick and mite infestations and provided insights into the inhibitory mechanisms of its phytochemicals.

## 1. Introduction

Sarcoptic mange, known as scabies in humans, is a highly contagious skin disease caused by *Sarcoptes scabiei*, a mite that burrows into the top layer of the skin (epidermis). These mites are astigmatids that belong to the Sarcoptinae family. They actively penetrate the outermost layer of the skin, known as the stratum corneum [1]. The adult mites mate, and the females lay eggs on the skin. Once hatched, larvae create small burrows, referred to as molting pouches, where they undergo molting and develop into nymphs, eventually becoming adults [2]. These parasites are found worldwide and can infect more than 150 different host species. Interestingly, they exhibit a surprising ability to be transmitted between different hosts, showcasing their epidemiological flexibility [3]. In animals, the disease can manifest as a mild infection. The common symptoms include itchy papules, redness (erythema), scales, and hair loss (alopecia). In chronic cases, hyperkeratosis (thickening of the skin) and/or the formation of crusts with discharge may occur [4,5]. Specific identification of this species depends on the host it infests. For example, in humans, it is *S. scabiei* var. *hominis*, whereas, in rabbits, it is referred to as *S. scabiei* var. cuniculi [6].

Sarcoptic mange is a highly contagious skin disease that spreads through direct skin-to-skin contact, contact with contaminated objects, and exposure to an infected environment inhabited by severely affected hosts [7,8]. Nymphs and female mites have a longer off-host survival period of up to 21 days than larvae and males, indicating the need for environmental application of biocides and repellents [9,10]. *S. scabiei* infection causes significant morbidity and mortality in wild and domestic mammals, leading to potential economic losses. Primary morbidity is associated with secondary bacterial infections caused by *Streptococcus pyogenes* and *Staphylococcus aureus* [11]. Rabbits infected with *S. scabiei* experience weight loss, reduced productivity, and compromised wool-fiber quality [12,13]. Additionally, affected rabbits may develop dermatitis, pyoderma, eczema, and urticaria [12].

Ticks are of great economic significance as pests in the global livestock industry, affecting cattle and other domestic species [14]. According to FAO [15], over 80% of the global cattle population is infected with ticks. Among these pests, *Rhipicephalus* (*Boophilus*) *microplus* is of major concern in tropical and subtropical regions. The impact of *R. microplus* infestation includes reduced milk production and weight gain, increased mortality rates, hide damage, disease morbidity, control costs, and transmission of tick-borne pathogens, such as *Babesia bigemina*, *B. bovis,* and *Anaplasma marginale*. The global financial losses caused by tick infestation are estimated to be approximately USD 14,000–18,000 million. Moreover, the livestock industry in India and Pakistan spends approximately USD 498.7 million annually on tick and TBD (tick-borne disease) control [16].

Currently, scabies and *R. microplus* are primarily managed by chemical acaricides. These acaricides consist of active components categorized as macrocyclic lactones, organophosphates, formamidines, synthetic pyrethroids, phenyl pyrazoles, and growth inhibitors [17]. Unfortunately, excessive, and sometimes inappropriate, use of these acaricides against ticks has led to the emergence of resistant tick populations [18,19]. Developing new synthetic compounds is a costly and time-consuming endeavor, underscoring the need for plant-based alternatives to effectively control tick and mite infestation [20,21,22].

Green tea, scientifically known as *Camellia sinensis*, holds a prominent position among beverages worldwide and has deep cultural significance in China and Japan. The numerous health benefits of *C. sinensis* are primarily associated with its polyphenol content. These polyphenols consist mainly of catechins and derivatives such as (−)-epigallocatechin-3-gallate (EGCG), (−)-epicatechin, (−)-epigallocatechin, (−)-epicatechin gallate, and (−)-gallocatechin gallate [23]. Studies have shown that *C. sinensis* exhibits anticancer, antitrypanosomal, and anti-*Plasmodium* properties [24,25]. Despite extensive research on the pharmacological aspects of *C. sinensis*, no studies have been conducted to explore its acaricidal activity.

Glutathione transferase (GST), classified under the EC 2.5.1.18 enzyme superfamily, plays a pivotal role in cellular detoxification processes. These enzymes facilitate the conjugation of reduced glutathione (GSH) with a wide range of endogenous and exogenous electrophilic compounds. By doing so, they protect cells from oxidative damage, contributing to overall cellular health.

In ticks, GST enzymes are believed to support tick survival by neutralizing toxins and aiding in the detoxification of various substances. This detoxification capacity is particularly relevant as overexpression of GST has been associated with drug resistance [26,27]. Several studies have observed an upregulation in the transcription of GST genes and an increase in GST enzyme activity in ticks when exposed to both endogenous and exogenous compounds [28,29].

Given these critical biological functions, GSTs emerge as promising targets for the development of novel acaricidal chemotherapeutic drugs. By selectively inhibiting GST activity, it is possible to disrupt detoxification mechanisms in ticks and mites, potentially rendering them more susceptible to acaricides. This approach holds promise in addressing challenges associated with mite and tick infestations and drug resistance, ultimately contributing to more effective mite and tick control strategies.

The pivotal role of GST enzymes in detoxification processes and their association with drug resistance make them attractive candidates for targeted acaricidal drug development, offering new avenues for parasitic Acari control and management.

The current study aimed to determine the in vitro efficacy of *C. sinensis* plant extract against ticks and mites. Natural products are a mix of different phytochemicals. Testing the relative contribution of individual compounds is difficult and inefficient given complex mixtures. We selected the most abundant compounds (Table 1) for the in silico evaluation of inhibitory interactions with tick and mite proteins. This provides a foundation for future bioassays to evaluate the insecticidal role of individual compounds.

## 2. Materials and Methods

### 2.1. Plant Extract Preparation

*C. sinensis* aerial materials were collected from the Jalala region in Mardan, Khyber Pakhtunkhwa (coordinates: 34.3345° N, 71.9075° E). The collected plant materials were inspected for any physical damage and then rinsed in running tap water to remove surface debris. The leaves were then submitted to the herbarium of the Department of Botany, Abdul Wali Khan University Mardan (AWKUM) and were issued the accession number Awkum.Bot.917. They were then kept in a shady place at room temperature, away from sunlight, for air drying.

After 15 d of air drying, the plant material was ground to form a coarse powder using a plant grinder (YUEYUEHONG Model: HC-3000A Zhejiang, China). Powdered material (100 g) of each plant was then soaked in 1000 mL of 80% ethanol and agitated for 48 h using an orbital shaker (labForce Model 1165U07, Thomas Scientific, Swedesboro, NJ, USA) at 300RPM. The agitated solution was then reduced to a concentrated solution using a rotary evaporator (BUCHI Rotavapor Model: R-300 Flawil, Switzerland) at 40 °C under vacuum to remove ethanol. The solution was further concentrated in a water bath (Model WTB15, Memmert GmbH & Co. KG, Schwabach, Germany) at 45 °C until a very high concentration of the extract less than 10% of the original volume remained. The concentrated extract was used as a stock solution at different concentrations. 

Different concentrations of 0.25, 0.5, 1, and 2 g/mL were prepared according to the procedure described by [13] for the acaricidal contact bioassay against mites and 2.5, 5, 10, 20, and 40 mg/mL concentrations for the acaricidal bioassay against ticks [30].

### 2.2. Mite Collection and Identification

Mites were collected from rabbits kept on rabbit farms at the Abdul Wali Khan University Mardan (AWKUM). Hay was provided as bedding to the rabbits on the farm, which was changed daily. The rabbits were first diagnosed for any signs of mange, and upon confirmation, skin scraps were taken using the procedure described by [13], and the rabbits were treated immediately. The skin scraps were then made into slides and observed under a microscope for *S. scabiei*.

### 2.3. Tick Collection and Incubation

Engorged adult female ticks were collected from the ground and cattle bodies in different locations near AWKUM in Mardan, Khyber Pakhtunkhwa, Pakistan. Standard tick identification keys were utilized to morphologically identify these ticks as *R*. (*B*.) *microplus* under a stereo zoom microscope [31]. The engorged ticks were then transported to the parasitology lab, AWKUM, and incubated to lay the eggs. The eggs were then incubated to hatch into larvae for larval assays in an incubator (BIOBASE Model: BJPX-H50IV Shandong, China). Adult ticks and larvae were then used in AIT and LPT.

### 2.4. Contacts Bioassay for Mites

Scabies-infested rabbits were used for isolating mites in the current study. Skin scraps were taken from the rabbit adhering to the established animal-care guidelines [32]. The infested skin was briefly cleaned and then scraped into a micro-Petri plate using a sterile surgical blade until the skin appeared red. The Petri plates containing skin scraps were incubated at 37 °C for 30 min to allow mites to emerge from the skin scraps. Ten mites were introduced into a Petri plate using a fine needle, and then 0.5 mL of samples of the plant extract were directly added onto the mites in the Petri plate. The procedure was performed in triplicate for each extract concentration.

### 2.5. Adult Immersion Test (AIT)

Completely engorged adult female ticks were used in the present study. The adult ticks were first rinsed in distilled water to remove any debris from their skin, and their weights were recorded. Ten (10) ticks were then dipped for 2–3 min at a concentration of the extract and then incubated for two days. This procedure was repeated for each extract concentration. Three replicates were performed for the study on separate days using a fresh new concentration of the extract for each replicate. Data were recorded for the total weight of eggs laid and tick mortality. The calculation of the percentage inhibition of oviposition (% IO) was performed using the following formula:

$$Percent\;inhibition\;of\;oviposition\;(\%IO)=\frac{Egg\;laying\;Index\left(control\right)-Egg\;laying\;Index\left(treated\right)}{Egg\;laying\;Index\;control}\times 100\%$$

The egg-laying index is calculated by dividing the mean weight of eggs laid by the mean weight of engorged females. 

### 2.6. Larval Packet Test (LPT)

Newly hatched larvae were used in this experiment. Whatman no. 1 filter paper was immersed in a 0.6 mL extract concentration and then dried in an incubator at 37 °C. The filter paper was then made into a pouch by bending it and taping its sides with adhesive tape, with the upper side left open. Then, 100 larvae were carefully placed in the pouch, making a packet of larvae. The packet was then incubated at 28 ± 1 °C in a bio-oxygen demand incubator (BOD incubator) (BIOBASE Model: BJPX-B100 Shandong, China). Larvae were inspected for mortality at 24 and 48 h. Larvae that did not respond to light or had no appendage movement when teased with a needle were considered dead. This assay was also performed in triplicates.

### 2.7. Selection of Phytochemicals

Following a thorough examination of the current literature using Google Scholar (https://scholar.google.com/, accessed on 25 June 2023, Keywords; “*Camellia sinensis*” AND “phytochemistry” OR phytochemicals AND “Pakistan” OR “India”) and PubMed (https://pubmed.ncbi.nlm.nih.gov/advanced/, accessed on 25 June 2023, Keyword; (Camellia sinensis [Title/Abstract]) AND (Pakistan [Title/Abstract]) AND (Phytochemistry) OR (phytochemicals) OR (GC-MS)), the *C. sinensis* phytochemicals were chosen. Articles that studied the phytochemical content of *C. sinensis* from Pakistan, India, or other related climates, utilizing GC-MS, TLC, and HPLC analytical methodologies were specifically chosen [33,34,35,36]. Ten phytochemicals were chosen from extracts of *C. sinensis* (L) because of their ability to inhibit the *R. microplus* and *S. scabiei* glutathione transferase (GST) protein. These phytochemicals, which possess anti-inflammatory, antiseptic, and astringent properties, were identified through an extensive review of the existing literature. The corresponding structures of the ligands targeting GST proteins were acquired from the PubChem-NCBI database in the structure data format (SDF). Subsequently, these structures were converted into the protein databank (PDB) format using PyMOL for further analysis.

### 2.8. Preparation of Protein by Homology Modeling

The RCSB’s (Research Collaboratory for Structural Bioinformatics) protein database (PDB) does not contain the available three-dimensional (3D) structures of the drug targets selected for this study. Therefore, homology modeling was used to obtain the 3D structures. The primary structures of *S. scabiei* glutathione transferase (Uniprot accession no: Q8I9R9) and *R. microplus* glutathione transferase (Uniprot accession no: O97117) were obtained in FASTA format from the UniProt Knowledgebase (UniProt KB database). To predict the 3D structure of SsGST and RmGST proteins, the SWISS-MODEL protein modeling server was employed [37]. This server generates homology models by performing a target-template sequence alignment using the BLASTp and HHBlits programs and searching through template structures in the Protein Data Bank (PDB) [38] and SWISS-MODEL Template Library (SMTL) repositories. The top-ranked alignments of the templates are compared using the global model quality estimate (GMQE) and quaternary structure quality estimate (QSQE) to generate sets of descriptive three-dimensional structures, sequence dissimilarity, and quaternary protein structure information. The QMEAN values were utilized to predict 3D protein structures, taking into account modeling errors and quality estimation. These predicted protein structures are then assessed for stereochemical quality using the SAVES v6.0 server (https://saves.mbi.ucla.edu/, accessed on 27 June 2023). Additionally, the Ramachandran plot, which plots Ψ versus Φ conformational angles of the 3D macromolecule, is used to measure the torsion angles of Cα (ideal) -N-Cβ (obs). To predict the active site amino acids, the CastP calculation server (http://sts.bioe.uic.edu/castp/calculation.html, accessed on 28 June 2023) is employed, which calculates the delineating surface area and surface volume of the 3D protein structure [39].

### 2.9. Preparation of Modeled Proteins and Ligands for Docking

To facilitate docking, homology-modelled SsGST and RmGST proteins were prepared. Gasteiger charges and polar hydrogens were added, and nonpolar and polar hydrogen atoms were merged by introducing partial charges using AutoDock Vina 4.2 [40]. To obtain structural information about bioactive compounds isolated from the leaves, fruit, and bark of *C. sinensis*, the relevant literature by [41] was reviewed. The 3D structures of these compounds were retrieved from the PubChem website (https://pubchem.ncbi.nlm.nih.gov/, accessed on 30 June 2023). Prior to docking, rotatable bonds were determined and nonpolar hydrogens were combined with polar hydrogen atoms for both phytochemical structures and conventional drugs for comparison purposes.

The docking process was conducted using AutoDock Vina software (version 1.1.2). Grid dimensions of 40 × 40 × 40 Å (grid size) with a grid-point spacing of 1.000 Å were employed for both proteins. The X, Y, and Z coordinates (grid centers) varied based on the specific receptor. The exhaustiveness parameter was set to a default value of eight (8) for all docking runs. The binding energy/affinity between the ligand and protein was calculated using the search algorithm within the AutoDock Vina software package. Following the completion of the docking runs, multiple binding modes representing different conformations of the ligands were obtained, along with their respective binding energy/affinity values. The most stable conformation, characterized by the lowest binding energy/affinity, was selected as the pose and utilized for postdocking analysis using BIOVIA Discovery Studio 2021. 

### 2.10. Molecular Dynamics Simulation Analysis 

Investigating the dynamic motion of atoms is crucial to understanding the stability and functionality of protein complexes. Molecular dynamics simulation plays a significant role in this regard [42]. To perform dynamic simulations of the docking complex, we utilized the iMODS server, which is specifically designed for this purpose [43]. The iMOD server (iMODS) (http://imods.chaco nlab.org, accessed on 3 July 2023) was employed for conducting molecular dynamics (MD) simulations of the protein–ligand complexes. These simulations allowed us to assess the stability and molecular motion of docked complexes.

Utilizing the iMOD server (iMODS), we performed molecular dynamics simulations to analyze the structural dynamics of the docking complexes and determine molecular motion. Various parameters, such as deformability, B-factor, eigenvalues, variance, covariance map, and elastic network, were employed to evaluate the stability of the two protein–ligand complexes. The input files used for the simulations were docked PDB files that were uploaded to the iMODS server with default parameter settings.

### 2.11. Statistical Analysis

All statistical analyses were performed using R (version 4.3) running in RStudio (version 2023.06.1). The data were first arranged in Microsoft Excel (v. 2302) and imported into the R working environment for further statistical analysis. Descriptive statistics of the data were calculated and are presented as mean ± standard deviation. The significance difference between the different concentrations was calculated using a one-way analysis of variance (ANOVA) followed by the Tukey honestly significance difference (HSD) test. Furthermore, 50% and 90% lethal concentrations and lethal times (LC and LT) were calculated in RStudio using the ecotox package and all the data were graphically presented using the ggplot2 and ggpubr R packages.

## 3. Results

### 3.1. Mites Contact Bioassay

The efficacy of the *C. sinensis* leaf extract against *S. scabiei* var. *cuniculi* mites was evaluated in vitro by measuring mean mortality rates at various concentrations and time intervals. Permethrin and distilled water were included as control groups for comparison (Table 2).

**Figure 1:**
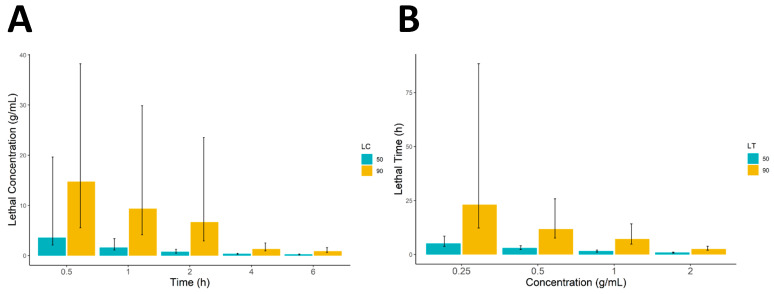
(**A**,**B**) represents the lethal concentrations and lethal time (LC_50_, LC_90_ and LT_50_, LT_90_), whereas the error bars represent the lower and upper confidence limits as 90% confidence intervals.

Table 3 presents the results of the lethal concentration (LC) calculations. As the time interval increased, the LC_50_ and LC_90_ values decreased, indicating a higher potency of the extract. At 6 h, the LC_50_ and LC_90_ values were 0.247 g/mL and 0.34 g/mL, respectively (Table 3, Figure 1A). Similarly, as the concentration increased, the LT_50_ and LT_90_ values decreased, indicating a faster onset of mortality (Table 4 and Figure 1B).

The results demonstrated the concentration- and time-dependent effects of *C. sinensis* leaf extract on *S. scabiei* mites. Higher concentrations and longer exposure times were associated with reduced LC_50_, LC_90_, LT_50_, and LT_90_ values, indicating increased efficacy in inducing mortality. The slope values reflect the steepness of the dose-response or dose-time curves, while the intercept values represent the baseline response.

### 3.2. Adult Immersion Test

The percentage of oviposition inhibition (% IO) of the ticks was calculated to assess the efficacy of different concentrations of extracts derived from *C. sinensis* in controlling tick populations. The % IO values provided insight into the ability of the extract to inhibit tick egg hatchability at varying concentrations and time intervals. The highest % IO was observed with the highest concentration of *C. sinensis* extract (40 mg/mL), showing a remarkable inhibition rate of 46.071 ± 7.797%. As the concentration decreased, the % IO values also declined, with 20 mg/mL, 10 mg/mL, 5 mg/mL, and 2.5 mg/mL resulting in % IO values of 36.44 ± 4.99%, 25.32 ± 2.995%, 15.468 ± 6.065%, and 4.825 ± 2.828%, respectively, as shown in Table 5. These findings highlight the potential of the *C. sinensis* extract to inhibit egg-hatching ability. Higher concentrations of the extract resulted in an increased % IO, indicating greater efficacy in controlling tick populations (Table 5).

### 3.3. Larval Packet Test

**Figure 2:**
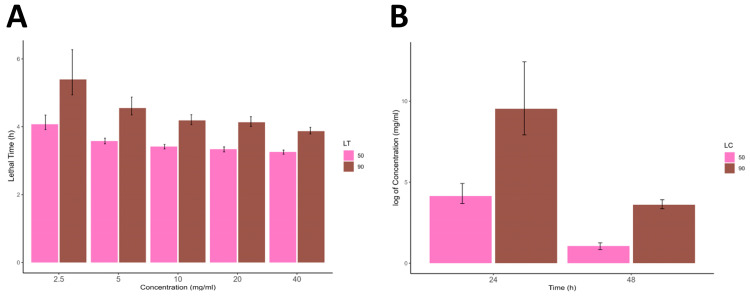
(**A**,**B**) represents the lethal concentrations and lethal time (LC_50,_ LC_90_, and LT_50,_ LT_90_), whereas the error bars represent the lower confidence limit and upper confidence limit at 90% confidence intervals.

The effects of different concentrations of *C. sinensis* extract on tick mortality were evaluated at 24 h and 48 h intervals. The control group consisted of ticks treated with deltamethrin and distilled water. The mean values and standard deviations (SD) of the measurements are listed in Table 5. The LC_50_–LC_90_ values and LT_50_ LT_90_, along with their respective 95% confidence limits (CL), were calculated for different time intervals (24 and 48 h), and various concentrations (2.5, 5, 10, 20, and 40 mg/mL) and are presented in Table 6 and Table 7. At the 48 h mark, the LC50 value was 2.906 mg/mL (95% CL:2.300–3.505), while the LC90 value was 36.725 mg/mL (95% CL:28.999–49.837), as shown in Table 6. LT50 values were calculated for different concentrations of the extract. At 2.5, 5, 10, 20, and 40 mg/mL, the LT50 values were 58.590, 35.788, 30.406, 28.078, and 25.913 h, respectively (Table 7, Figure 2A). The larval packet test results showed that with an increase in concentration or time, the LT and LC values decreased (Figure 2B).

### 3.4. Homology Modeling

To analyze the structural arrangement of both proteins, 3D models were generated using the SWISS-Model online server. The SWISS-Model employs BLAST and HHblits to align the target sequences with previously characterized sequences and identify the most suitable template(s). The selected templates for the SsGST and RmGST proteins were 4q5q.1.A and 6gsv.1.A, respectively, with maximum sequence identities of 62.67% and 53.00% and coverages of 99% and 92%, respectively. The predicted model range was 219 and 223 amino acids for SsGST and RmGST, respectively. The root-mean-square deviation (RMSD) values for SsGST and RmGST, with their respective templates, were 0.067 and 0.084, respectively, indicating reasonable structural similarity.

**Figure 3:**
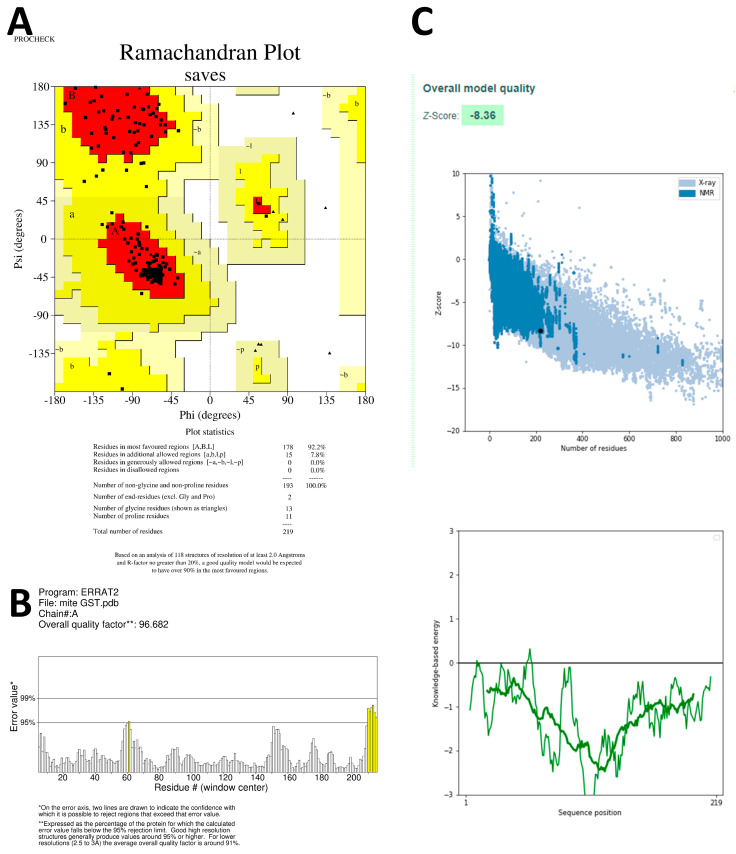
Validation plots and scores for the TrRosetta server’s predicted 3D structure of *Sarcoptes scabiei* glutathione transferase (SsGST) showing (**A**) the Ramachandran plot where the red, yellow, and black colors represent the most favorable, favorable, and disallowed regions, respectively; Phi and Psi bonds represent torsion angles that predict the possible conformation of the peptides; (**B**) represents the ERRAT’s overall quality factor values; and (**C**) represents the PROSA server’s Z score values.

**Figure 4:**
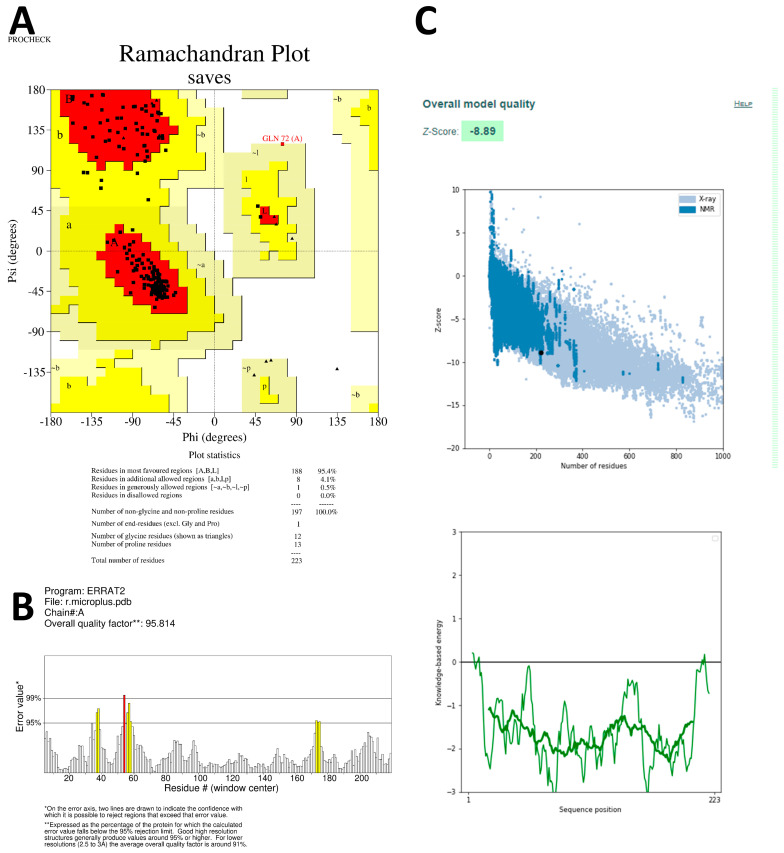
Validation plots and scores for the TrRosetta server’s predicted 3D structure of *Rhipicephalus microplus* glutathione transferase (RmGST) showing (**A**) the Ramachandran plot where the red, yellow, and black colors represent the most favorable, favorable, and disallowed regions, respectively; Phi and Psi bonds represent torsion angles that predict the possible conformation of the peptides; (**B**) represents the ERRAT’s overall quality factor value; and (**C**) represents the PROSA server’s Z score values.

The stereochemistry of both models was assessed using Ramachandran plots, which categorized amino acids into the core, additionally allowed, generously allowed, and disallowed regions. The Ramachandran plot for SsGST showed 92.2% amino acids in the core region (Figure 3A), while the plot for RmGST demonstrated 95.4% amino acids in the core region and one amino acid in the generously allowed region (Figure 4A). No amino acids appeared in the disallowed region of either protein. The quality of the overall protein structure was evaluated using z plots and ProSA. The z scores for both predicted models were −8.36 (Figure 3C) and −8.89 (Figure 4C), respectively, indicating acceptable overall quality comparable to NMR protein structures.

To further validate the 3D models, the ERRAT online server was used to validate. The overall quality factors calculated using ERRAT were 96.682 for SsGST and 95.814 for RmGST (Figure 3B and Figure 4B, respectively), confirming the validity of the predicted models. These results confirm the reliability of the predicted models for the SsGST and RmGST proteins.

### 3.5. Active Sites Prediction

**Figure 5:**
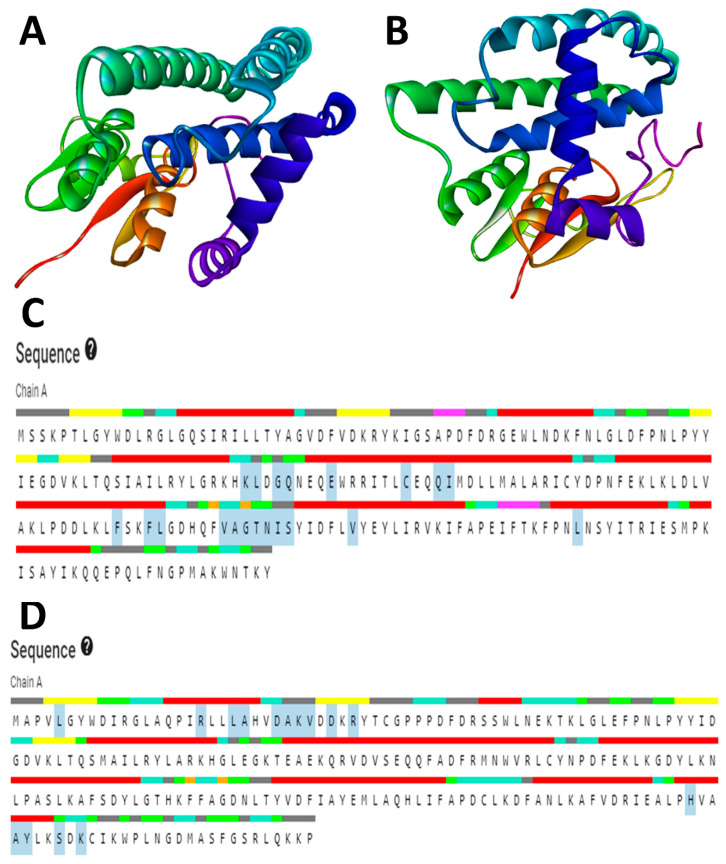
SWISS MODEL modelled 3D structures of (**A**) SsGST and (**B**) RmGST. (**C**,**D**) represents the CASTp server-predicted active sites of SsGST and RmGST respectively.

The model structure’s active site was analyzed through the utilization of the CASTp server, which also facilitated the determination of the amino acid residues present in the active site. The outcomes were subsequently visualized using PyMOL (Figure 5). The crucial step in drug or inhibitor design involves the identification and characterization of active site residues. According to the CASTp prediction, the active residues for the SsGST protein were identified as ARG80, TYR81, ARG84, ASP89, GLU93, TRP96, ARG97, ARG98, ILE99, THR100, GLU103, and TYR157 (Figure 5C). Similarly, for the RmGST protein, the active sites were determined to be ARG18, LEU21, ALA22, HIS23, ASP25, ALA26, LYS27, VAL28, ASP30, ARG32, HIS193, VAL194, ALA196, TYR197, SER200, LYS202, and CYS203 (Figure 5D).

### 3.6. Molecular Docking

**Figure 6:**
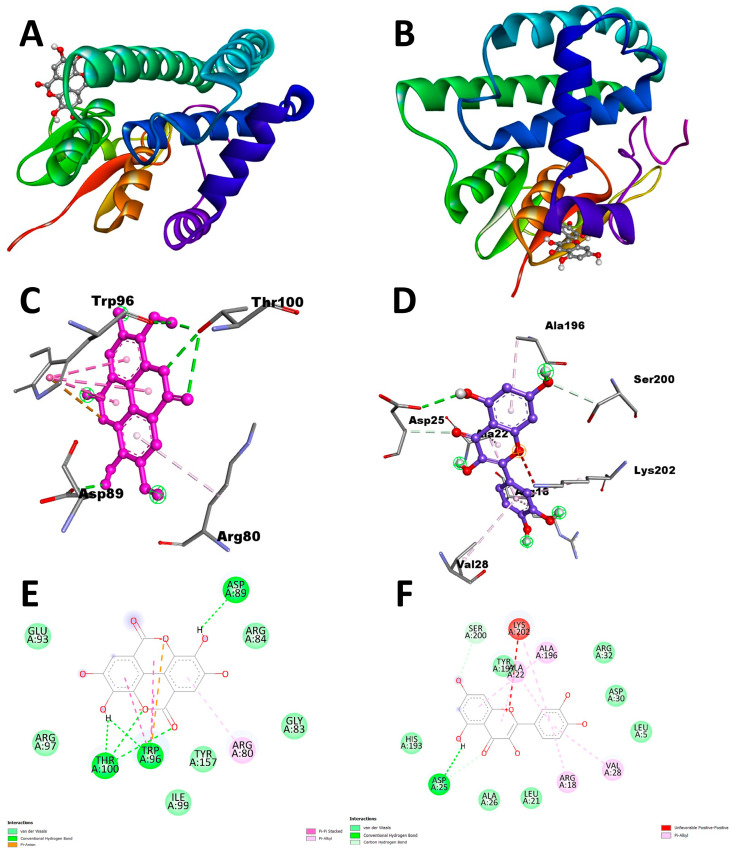
(**A**,**C**,**E**) show the complex three-dimensional structural interactions of the ellagic acid and SsGST and their corresponding 2D interactions; (**B**,**D**,**F**) show the highly complex 3D structural interactions of the quercitin and RmGST and their corresponding 2D interactions.

In this study, all ten phytochemicals exhibited significant inhibitory potential against the target proteins. Notably, ellagic acid, epigallocatechin gallate, kaempferol, and quercetin demonstrated the most promising results, targeting SsGST and RmGST proteins, respectively, with better binding scores than their respective controls (Table 8). Molecular docking analysis revealed that ellagic acid exhibited a strong binding affinity towards the SsGST protein, with a binding energy of −7.3 kcal/mol, surpassing permethrin (−6.7 kcal/mol), as shown in Figure 6A,C,E and Table 8. Ellagic acid formed hydrogen bonds with Thr-100 (three bonds), Trp-96 (one bond), and Asp-89 (one bond) residues of the SsGST protein. Furthermore, hydrophobic interactions were observed between ellagic acid and Trp-96 and Arg-80, indicating their potential as inhibitors of *S. scabiei* glutathione transferase. Among other ligands, epicatechin gallate also displayed a favorable binding energy (−6.8 kcal/mol) and amino acid interactions compared to permethrin (−6.7 kcal/mol).

In contrast, quercetin demonstrated a strong binding affinity for the RmGST target protein (Table 8, Figure 6D,F), with a docking score of −8.6 kcal/mol compared to deltamethrin (−8.2 kcal/mol). Quercetin formed a hydrogen bond with Asp-125 and established π-alkyl bonds with Arg-18, Val-28, Ala-196, and Ala-22, as shown in Figure 6B,D,F. In addition, a carbon–hydrogen bond was observed with Ser-200. These findings suggest that quercetin has the potential to inhibit RmGST and can serve as an antitick agent.

### 3.7. Molecular Dynamics Simulation Analysis 

**Figure 7:**
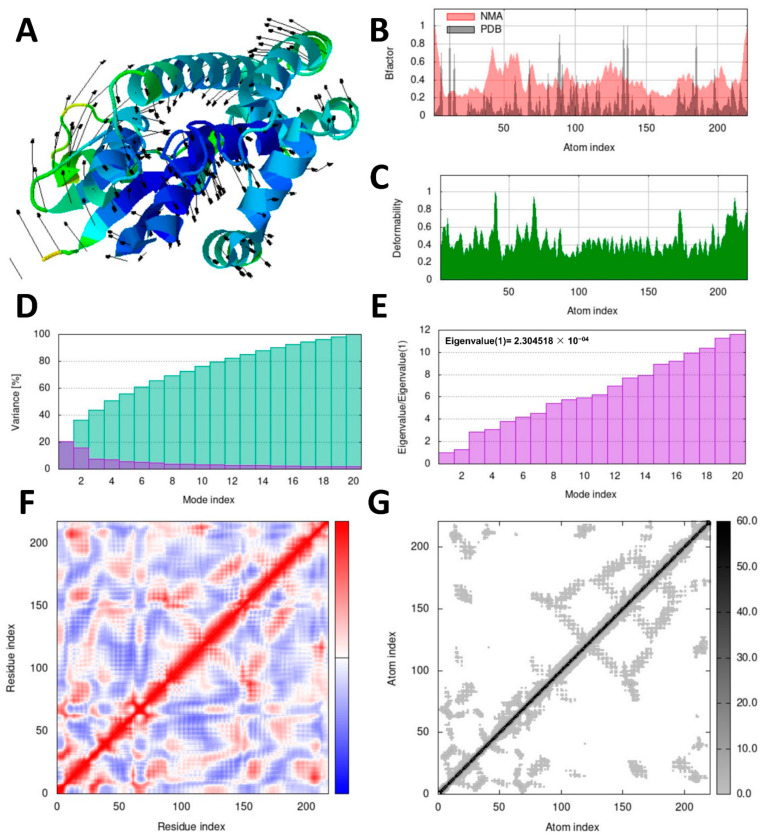
The iMOD server’s normal mode analysis (NMA) outputted the following plots for the ellagic acid–SsGST complex. (**A**) represents the NMA mobility, (**B**) B-factor, (**C**) deformation plot, (**D**) variance plot, (**E**) eigenvalues, (**F**) covariance-matrix plot (the anticorrelated, uncorrelated, and correlated states of atomic motion are represented by the blue, white, and red hues, respectively), and (**G**) elastic network model, where grey colors represent atom connections.

**Figure 8:**
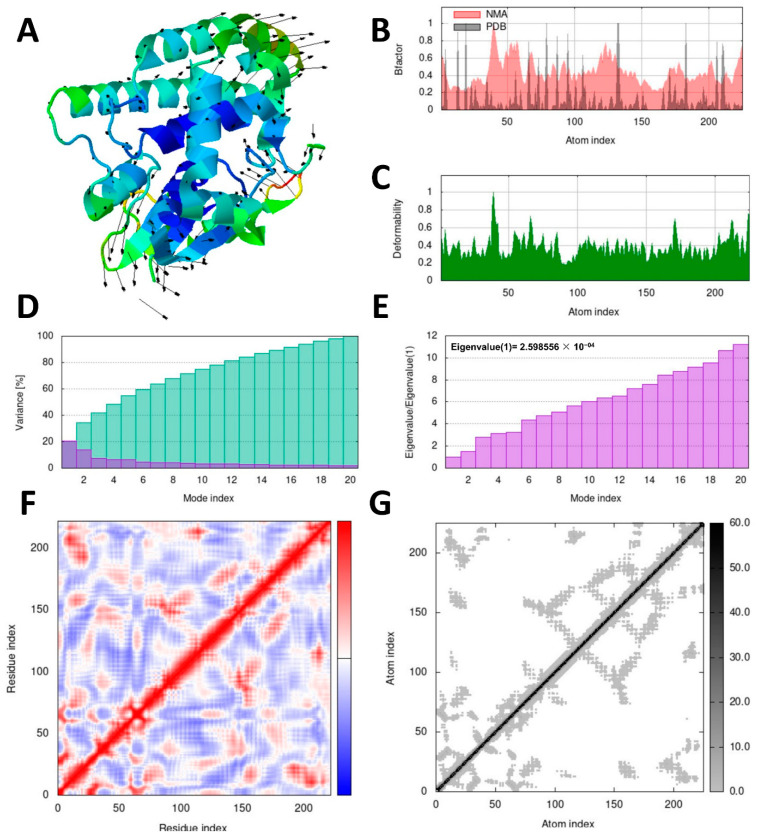
The iMOD server’s normal mode analysis (NMA) outputted the following plots for the quercitin–RmGST complex. (**A**) represents the NMA mobility, (**B**) B-factor, (**C**) deformation plot, (**D**) variance plot, (**E**) eigenvalues, (**F**) covariance-matrix plot (the anticorrelated, uncorrelated, and correlated states of atomic motion are represented by the blue, white, and red hues, respectively), and (**G**) elastic network model, where grey colors represent atom connections.

To assess the stability and dynamics of the docked complexes, molecular dynamics (MD) simulations were conducted using the iMOD server. Normal mode analysis (NMA) was employed to examine the slow dynamics and large-scale conformational fluctuations of the docked complexes, namely ellagic acid–SsGST and quercetin–RmGST, as shown in Figure 7 and Figure 8, respectively.

Deformability and B-factor profiles provide valuable information about the mobility and flexibility of docked proteins. The peaks observed in these profiles indicated regions of higher deformability, suggesting greater flexibility or mobility within these regions. The highest peaks in the profiles represent the regions with the most pronounced flexibility. Figure 7 and Figure 8 provide illustrations of the deformability and B-factor profiles for the ellagic acid–SsGST and quercetin–RmGST complexes, respectively. These profiles enable a comparison between the results obtained from normal mode analysis (NMA) and structures obtained from the Protein Data Bank (PDB). By examining these profiles, the regions in proteins that exhibit significant flexibility or undergo conformational changes can be identified. This information can be valuable for understanding the dynamic behavior of docked complexes and their potential impact on protein function and stability.

The eigenvalue and variance graphs reveal the characteristics of each normal mode with an inverse relationship between the eigenvalues and variances. The eigenvalue and variance graphs for the ellagic acid–SsGST and quercetin–RmGST complexes are presented in Figure 7E and Figure 8E, respectively. Additionally, a covariance matrix was provided for the docked complexes, representing anticorrelated motion (depicted in blue), uncorrelated motion (depicted in white), and correlated motion (depicted in red). This matrix provides information about the atomic interactions and their dynamics. Atomic connections were visualized using elastic springs in a string-model representation (Figure 7F and Figure 8F), along with a plot matrix where the connections are depicted as grey dots (Figure 7G and Figure 8G).

## 4. Discussion

The use of plant extracts for pest and disease control has gained attention because of their natural degradation properties [44]. Despite their advantages, biopesticides represent a small portion of the pesticide market [45]. However, the biopesticide sector has experienced significant growth in recent years, with an annual growth rate predicted to surpass that of chemical pesticides [45,46].

This study focused on investigating the acaricidal efficacy of *C. sinensis* extract as a potential source for developing herbal acaricides and identifying bioactive compounds against ticks and mites. Ticks and mites were treated with different concentrations of the ethanol extracts. Previous studies have explored the acaricidal properties of various herbs against mites and ticks [13,30,47,48]. *C. sinensis* has been reported to possess antioxidant [49], antibacterial [50], anti-inflammatory, and antihistaminic properties [51], as well as insecticidal properties [52], whereas no acaricidal potential of the plant has been documented. The plant extract has resulted in greater mortality in mites and comparable mortality in ticks as compared to the positive control permethrin and deltamethrin. The decrease in the chemical acaricides toxicity can be due to the acaricidal resistance by these mites and ticks. In a study by Gu, et al. [53], the *Ailanthus altissima* bark extract has resulted in similar results with the extract having more significant acaricidal potential against *S. scabiei* and *Psoroptes scabiei* compared to the available chemical acaricide fenvalerate. The findings of this study are consistent with previous research of Seddiek, et al. [54]. Moreover, *Dodonaea angustifolia*, *Eucalyptus globulus*, *Millettia ferruginea*, and *Euphorbia abyssinica* plant extracts have been found to have acaricidal potential against these mites [52]. 

Molecular docking studies have been widely utilized to predict ligand–target interactions and gain insights into the biological activity of natural products. These studies also provide clues regarding the mechanisms of action and binding modes within the active sites of enzymes [55]. In this study, ten representative compounds from *C. sinensis* were selected for docking analyses against two target proteins: *S. scabiei* glutathione transferase (UniProt accession no: Q8I9R9) and *R. microplus* glutathione transferases (UniProt accession no: O97117).

Docking analysis with *S scabiei* glutathione transferase revealed that, among the ten compounds, ellagic acid exhibited strong interactions with several amino acid residues through hydrogen bonds (Thr-100, Trp-96, Asp-89) and hydrophobic interactions (Trp-96 and Arg-80), with a docking score of −7.3 kcal/mol. Epicatechingallate, epigallocatechingallate, epicatechin, quercetin, caffeoylquinicacid, kaempferol, catechin, gallicacid, and theanine also displayed docking scores, suggesting their potential involvement in the antimite activity of *C. sinensis* through interactions with the target protein.

In the antitick docking study, the ten compounds were docked with *R. microplus* glutathione transferases, and they exhibited docking scores ranging from −5.2 to −8.82 kcal/mol. Quercetin displayed the highest score against RmGST protein, followed by kaempferol, catechin, epigallocatechin gallate, epicatechin, caffeoylquinic acid, epicatechin gallate, ellagic acid, gallic acid, and theanine. Molecular dynamics simulations confirmed the low deformability of the docked proteins, supporting the validity of the in silico-predicted acaricide. Ellagic acid and quercetin have been reported to have antibacterial, antiviral, antimalarial, antiparasitic, antioxidant, and anti-inflammatory properties in previous studies [56,57,58,59]. The GST from *R. microplus* has also been reported to be inhibited by several other compounds such as anonaine from *Annona crassiflora* [60], norapoatropine, and 7-Hydroxyhyoscyamine from *Datura innoxia* [48].

Overall, this study provides computational evidence for the potential inhibition of SsGST and RmGST proteins. Further research should focus on evaluating the clinical efficacy of these compounds, which could contribute to the development of novel resources for managing the Acari species.

## 5. Conclusions

The acaricidal activity of *C. sinensis* against *R. microplus* and *S. scabiei* mites has been demonstrated in vitro, showing high larvicidal and adulticidal activities. Quercetin and ellagic acid, two bioactive compounds found in *C. sinensis*, have been identified as inhibitors of SsGST and RmGST protein enzymes. In silico studies have provided insights into the mechanisms of inhibition, indicating that quercetin and ellagic acid interact with the active site residues of RmGST and SsGST through hydrogen bonds and hydrophobic contacts. These findings suggest that quercetin and ellagic acid have the potential to be developed into new acaricidal drugs. Further studies are needed to evaluate their effects on adult stages and to assess their acaricidal activity under in vivo conditions.

## Figures and Tables

**Table 1:** Selected compounds from *C. sinensis* plant for its in silico screening against ticks’ and mites’ protein.

Compounds	PubChem CID
Caffeoylquinic acid	5359940
Catechin	9064
Ellagic acid	5281855
Epicatechin	72276
Epicatechin gallate	367141
pigallocatechin gallate	65064
Gallic Acid	370
Kaempferol	5280863
Quercetin	5280343
Theanine	439378

**Table 2:** The mean mortality ± standard deviation of the mortalities of *S. scabiei* var. *cuniculi* by *Camellia sinensis* leaf extract in vitro.

Plant	Concentration (g/mL)	n	Mean Mortality ± Its Standard Deviation				
			0.5 h	1 h	2 h	4 h	6 h
Camellia sinensis	2	3	2.667 ± 0.577 ^a^	5.667 ± 0.577 ^a^	7.333 ± 0.577 ^a^	10 ± 0 ^a^	10 ± 0 ^a^
	1	3	1.667 ± 0.577 ^ab^	3.667 ± 0.577 ^b^	5.333 ± 0.577 _b_	7.667 ± 0.577 ^b^	9 ± 0 ^b^
	0.5	3	0.333 ± 0.577 ^c^	1.667 ± 0.577 ^cd^	3.667 ± 0.577 ^cd^	6 ± 0 ^c^	7.333 ± 0.577 ^c^
	0.25	3	0 ± 0 ^c^	1 ± 1 ^cd^	2.667 ± 0.577 ^d^	4 ± 0 ^d^	5.333 ± 0.577 ^d^
Control Group	Permethrin 5% (*w/v*)	3	0.667 ± 0.577 ^bc^	2.667 ± 0.577 ^bc^	4.333 ± 0.577 ^bc^	7.333 ± 0.577 ^b^	9 ± 0 ^b^
	Distilled Water	3	0 ± 0 ^c^	0 ± 0 ^d^	0 ± 0 ^e^	0 ± 0 ^e^	2 ± 0 ^e^

Means with no similar letters in the superscript in the same column are significantly different by Tukey’s HSD test at the 5% level of significance (*p* < 0.05).

**Table 3:** The calculated lethal concentrations were responsible for 50% and 90% *S. scabiei* mortality at various time intervals for the *Camellia sinensis* leaf extract.

Time (h)	LC_50_ (g/mL)	95% Confidence Limits		LC_90_ (g/mL)	95% Confidence Limits		Slope ± SE	Intercept ± SE	Chi Square	*p*-Value
		LCL	UCL		LCL	UCL				
0.5	3.591	2.157	19.667	14.764	5.526	38.241	2.087 ± 0.627	−1.159 ± 0.172	3.719	0.959
1	1.625	1.126	3.39	9.366	4.131	29.872	1.685 ± 0.404	−0.355 ± 0.131	3.783	0.957
2	0.792	0.519	1.296	6.684	2.94	23.546	1.384 ± 0.363	0.14 ± 0.13	1.526	0.999
4	0.363	0.24	0.476	1.335	0.951	2.545	2.266 ± 0.444	0.997 ± 0.176	3.504	0.967
6	0.247	0.132	0.34	0.88	0.644	1.618	2.323 ± 0.52	1.41 ± 0.223	1.572	0.999

LC: lethal concentration, LCL: lower confidence limit, UCL: upper confidence limit, SE: standard error.

**Table 4:** The calculated lethal time was responsible for 50% and 90% *S. scabiei* mortality at varying concentration intervals for *Camellia sinensis* leaf extract.

Concentration (g/mL)	LT50 (h)	95% Confidence Limits		LT90 (h)	95% Confidence Limits		Slope ± SE	Intercept ± SE	Chi Square	*p*-Value
		LCL	UCL		LCL	UCL				
0.25	5.095	3.752	8.482	23.102	12.289	88.36	1.952 ± 0.377	−1.38 ± 0.207	4.727	0.981
0.5	3.004	2.348	4.035	11.809	7.624	25.842	2.156 ± 0.344	−1.03 ± 0.176	2.875	0.998
1	1.595	1.19	2.077	7.149	4.801	14.227	1.967 ± 0.31	−0.399 ± 0.14	2.031	1
2	0.9	0.687	1.117	2.58	1.982	3.894	2.801 ± 0.423	0.129 ± 0.138	4.909	0.977

LT: lethal time, LCL: lower confidence limit, UCL: upper confidence limit, SE: standard error.

**Table 5:** The % mean values ± standard deviation for different concentrations of *Camellia sinensis* leaf extract on *R. (B.) microplus* larval mortality at 24 and 48 h, as well as inhibition of oviposition in adult female *R. (B.) microplus*.

Plant	Concentration (mg/mL)	n	Mean ± SD of Tick’s Mortality		Mean (%) ± SD of IO
			24 h	48 h	% IO
*Camellia sinensis*	40	3	43.667 ± 2.517 ^a^	90 ± 4 ^a^	46.071 ± 7.797 ^b^
	20	3	40 ± 1 ^ab^	80.667 ± 2.082 ^b^	36.44 ± 4.99 ^bc^
	10	3	34.667 ± 3.512 ^bc^	77.667 ± 1.528 ^b^	25.32 ± 2.995 ^cd^
	5	3	30 ± 2.646 ^c^	65 ± 4.583 ^c^	15.468 ± 6.065 ^de^
	2.5	3	19.333 ± 1.528 ^d^	42.333 ± 3.055 ^d^	4.825 ± 2.828 ^e^
Control Group	Deltamethrin 2.5% (*w/v*)	3	45.667 ± 2.082 ^a^	94.333 ± 3.512 ^a^	65.956 ± 4.908 ^a^
	Distilled Water	3	0 ± 0^e^	1.333 ± 1.528 ^e^	3.331 ± 2.989 ^e^

Means with no similar letters in superscript in the same column are significantly different by Tukey’s HSD test at the 5% level of significance (*p* < 0.05).

**Table 6:** The calculated lethal concentrations were responsible for 50% and 90% mortality at various time intervals of *Camellia sinensis* exposure to *R. (B.) microplus* larvae.

Time (h)	LC_50_ (mg/mL)	95% Confidence Limits		LC_90_ (mg/mL)	95% Confidence Limits		Slope ± SE	Intercept ± SE	Chi Square	*p*-Value
		LCL	UCL		LCL	UCL				
24	62.925	39.488	137.689	13,834.038	2750.593	251,503.950	0.547 ± 0.080	−0.984 ± 0.089	6.034	0.945
48	2.906	2.300	3.505	36.725	28.999	49.837	1.163 ± 0.089	−0.539 ± 0.088	16.772	0.210

LC: lethal concentration, LCL: lower confidence limit, UCL: upper confidence limit, SE: standard error.

**Table 7:** Lethal times for 50% and 90% mortality at various concentrations of *Camellia sinensis* against *R. (B.) microplus* larvae.

Concentration (mg/mL)	LT_50_ (h)	95% Confidence Limits		LT_90_ (h)	95% Confidence Limits		Slope ±	Intercept ±	Chi Square	*p* Value
		LCL	UCL		LCL	UCL				
2.5	58.590	50.096	76.953	219.609	139.423	527.624	2.233 ± 0.367	−3.948 ± 0.572	1.064	0.900
5	35.788	33.043	38.955	95.018	77.517	130.440	3.022 ± 0.353	−4.695 ± 0.545	2.513	0.642
10	30.406	28.342	32.427	65.596	58.010	77.914	3.838 ± 0.364	−5.691 ± 0.556	1.358	0.851
20	28.078	25.925	30.065	62.103	54.975	73.804	3.717 ± 0.368	−5.384 ± 0.559	0.639	0.959
40	25.913	24.218	27.463	48.000	44.204	53.479	4.787 ± 0.407	−6.766 ± 0.608	4.070	0.397

LT: lethal time, LCL: lower confidence limit, UCL: upper confidence limit, SE: standard error.

**Table 8:** Docking result of phytochemicals against *Sarcoptes scabiei* glutathione transferase (SsGST) and *R. microplus* glutathione transferase (RmGST) protein.

S. No	Plant Name	Compound Name	PubChem CID	Compound Structures	Docking Score (Kcal/mol) against SsGST	Docking Score (Kcal/mol) against RmGST
1	*Camilla sinensis*	Caffeoylquinic acid	5359940	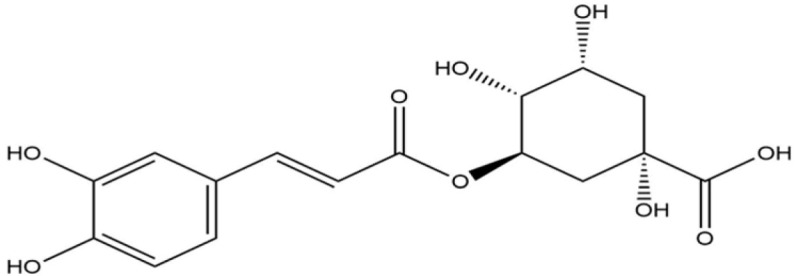	−6.2	−7.8
2		Catechin	9064	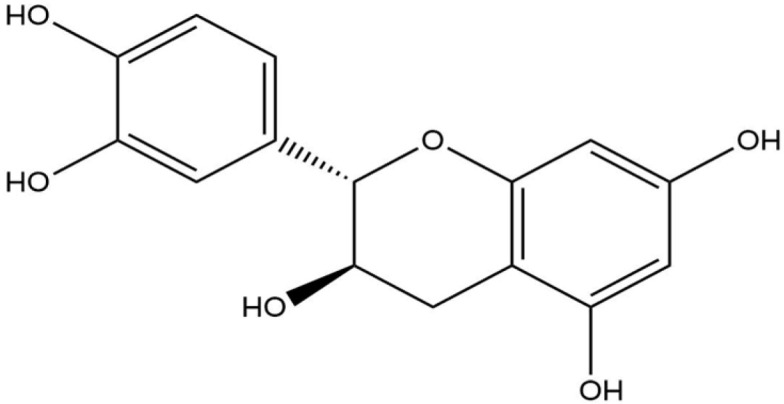	−5.9	−8.1
3		Ellagic acid	5281855	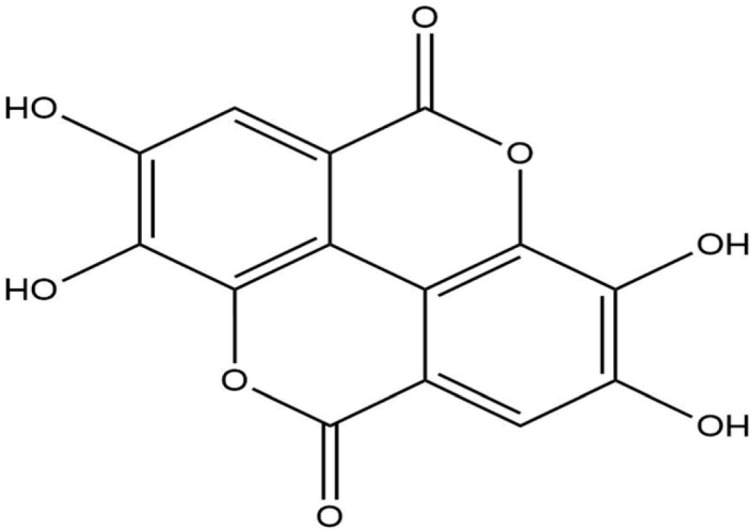	−7.3	−7.5
4		Epicatechin	72276	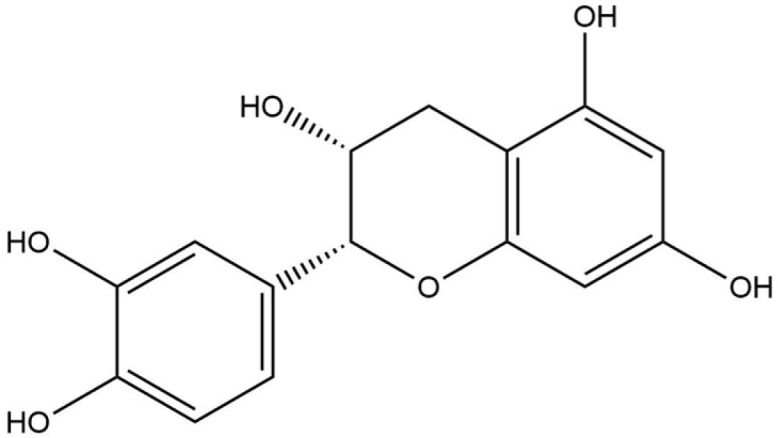	−6.3	−7.8
5		Epicatechin gallate	367141	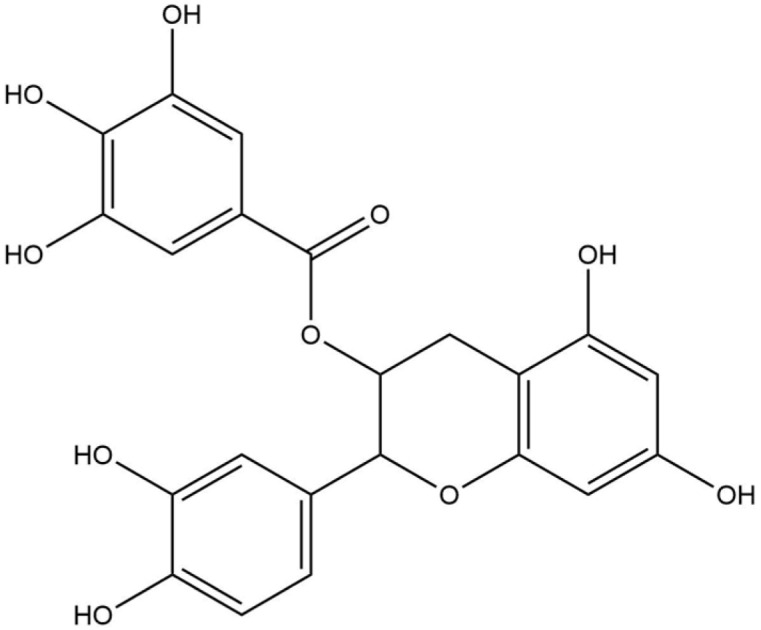	−6.8	−7.8
6		Epigallocatechin gallate	65064	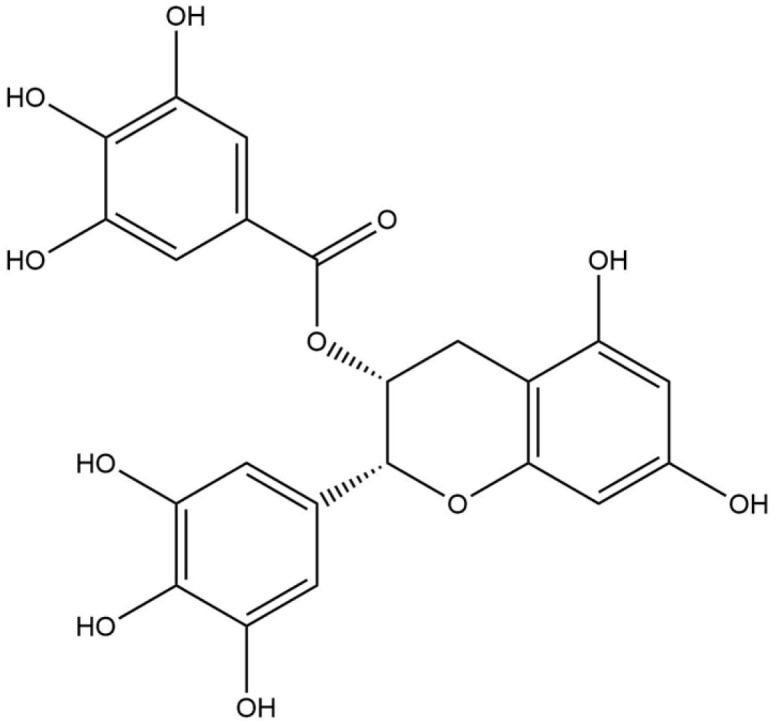	−6.7	−8.0
7		Gallic Acid	370	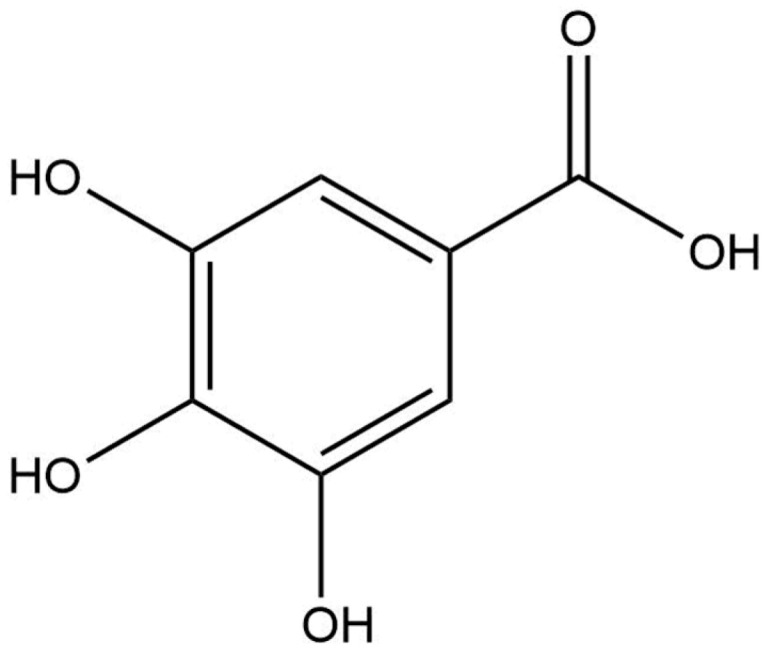	−5.3	−5.8
8		Kaempferol	5280863	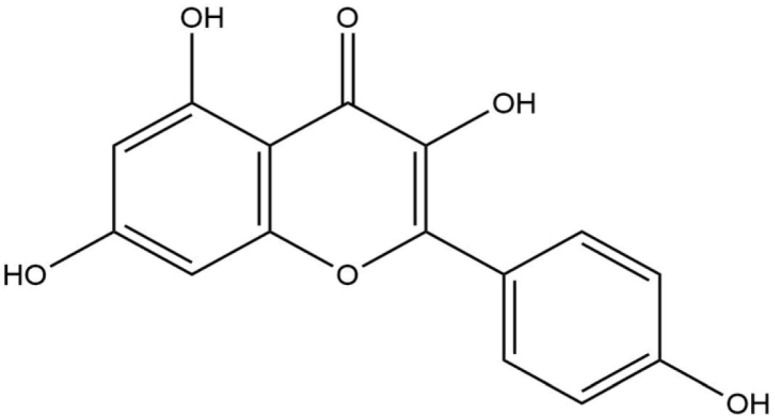	−6.2	−8.5
9		Quercetin	5280343	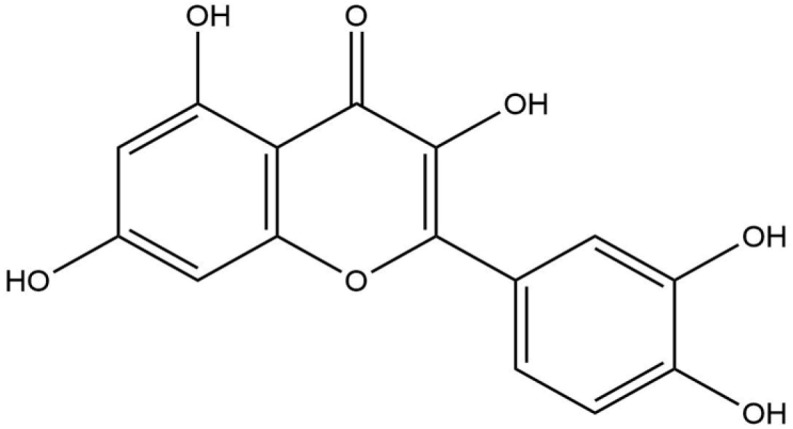	−6.2	−8.6
10		Theanine	439378	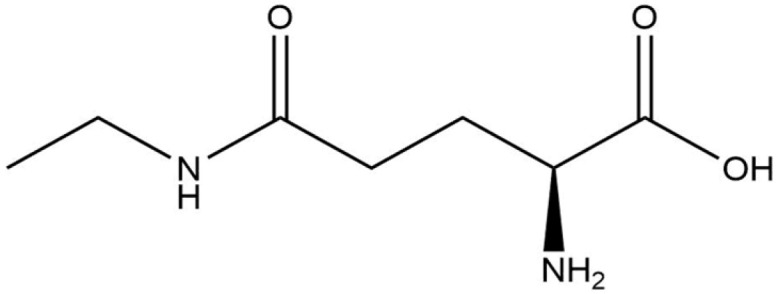	−4.5	−5.2
11	Standard Drug	Permethrin	40326	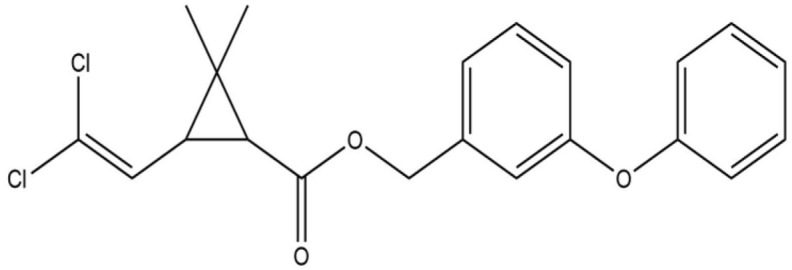	−6.7	-
12	Standard Drug	Deltamethrin	40585	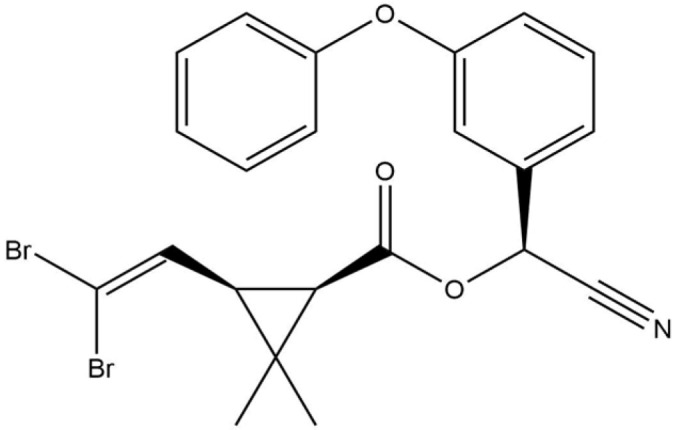	-	−8.2

## Data Availability

Not applicable.

## References

[1] Nardoni S., Mancianti F. (2022). Essential Oils against *Sarcoptes scabiei*. Molecules.

[2] Arlian L.G., Morgan M.S. (2017). A review of *Sarcoptes scabiei*: Past, present and future. Parasites Vectors.

[3] Moroni B., Rossi L., Bernigaud C., Guillot J. (2022). Zoonotic Episodes of Scabies: A Global Overview. Pathogens.

[4] Cardells J., Lizana V., Martí-Marco A., Lavín S., Velarde R., Rossi L., Moroni B. (2021). First description of sarcoptic mange in an Iberian hare (*Lepus granatensis*). Curr. Res. Parasitol. Vector-Borne Dis..

[5] Rahman M.M., Lecchi C., Fraquelli C., Sartorelli P., Ceciliani F. (2010). Acute phase protein response in Alpine ibex with sarcoptic mange. Vet. Parasitol..

[6] Dawod A., Fathalla S.I., Elkhatam A., Sheraiba N.I., Hammad M.A., El-Seedi H.R., Shehata A.A., Anis A., Fayed M.A.A., Osman N. (2023). Acaricidal Effects of Selamectin and *Ficus sycomorus* extracts on *Sarcoptes scabiei* Mites Infection in Rabbit. J. Curr. Vet. Res..

[7] Alasaad S., Rossi L., Heukelbach J., Pérez J.M., Hamarsheh O., Otiende M., Zhu X.Q. (2013). The neglected navigating web of the incomprehensibly emerging and re-emerging *Sarcoptes* mite. Infect. Genet. Evol..

[8] Bernigaud C., Fischer K., Chosidow O. (2020). The Management of Scabies in the 21st Century: Past, Advances and Potentials. Acta Derm. Venereol..

[9] Arlian L.G., Vyszenski-Moher D.L., Pole M.J. (1989). Survival of adults and developmental stages of *Sarcoptes scabiei* var. canis when off the host. Exp. Appl. Acarol..

[10] Fang F., Bernigaud C., Candy K., Melloul E., Izri A., Durand R., Botterel F., Chosidow O., Huang W., Guillot J. (2015). Efficacy assessment of biocides or repellents for the control of *Sarcoptes scabiei* in the environment. Parasites Vectors.

[11] Andriantsoanirina V., Guillot J., Ratsimbason M., Mekhloufi G., Randriamialinoro F., Ranarivelo L., Ariey F., Durand R. (2022). In vitro efficacy of essential oils against *Sarcoptes scabiei*. Sci. Rep..

[12] Seddiek S.A., Khater H.F., El-Shorbagy M.M., Ali A.M. (2013). The acaricidal efficacy of aqueous neem extract and ivermectin against *Sarcoptes scabiei* var. cuniculi in experimentally infested rabbits. Parasitol. Res..

[13] Khan A., Sohaib M., Ullah R., Hussain I., Niaz S., Malak N., de la Fuente J., Khan A., Aguilar-Marcelino L., Alanazi A.D. (2022). Structure-based in silico design and in vitro acaricidal activity assessment of *Acacia nilotica* and *Psidium guajava* extracts against *Sarcoptes scabiei* var. cuniculi. Parasitol. Res..

[14] Rodriguez-Vivas R.I., Jonsson N.N., Bhushan C. (2018). Strategies for the control of *Rhipicephalus microplus* ticks in a world of conventional acaricide and macrocyclic lactone resistance. Parasitol. Res..

[15] FAO (1984). Tick-Borne Disease Control: A Practical Field Manual.

[16] Rahman A., Kashif M., Nasir A., Idrees A., Jamil M., Elahi M.E., Qadir Z.A., Qasim M., Khan I., Aziz H. (2022). A Review of Tick and Tick Control Strategies in Pakistan. Pak. J. Med. Health Sci..

[17] Fantatto R.R., Gainza Y.A., Figueiredo A., Sorrechia R., Chagas A.C.d.S., Pietro R.C.L.R. (2022). The association of extracts of *Achyrocline satureioides* and the fungus *Beauveria bassiana* against the tick *Rhipicephalus microplus*. Exp. Appl. Acarol..

[18] Chandler D.J., Fuller L.C. (2019). A Review of Scabies: An Infestation More than Skin Deep. Dermatology.

[19] Klafke G., Webster A., Dall Agnol B., Pradel E., Silva J., de La Canal L.H., Becker M., Osório M.F., Mansson M., Barreto R. (2017). Multiple resistance to acaricides in field populations of *Rhipicephalus microplus* from Rio Grande do Sul state, Southern Brazil. Ticks Tick-Borne Dis..

[20] de Souza Chagas A.C., de Barros L.D., Cotinguiba F., Furlan M., Giglioti R., de Sena Oliveira M.C., Bizzo H.R. (2012). In vitro efficacy of plant extracts and synthesized substances on *Rhipicephalus* (*Boophilus*) *microplus* (Acari: Ixodidae). Parasitol. Res..

[21] Pasipanodya C.N., Tekedza T.T., Chatiza F.P., Gororo E. (2021). Efficacy of neem (*Azadirachta indica*) aqueous fruit extracts against *Sarcoptes scabiei* var. suis in grower pigs. Trop. Anim. Health Prod..

[22] Rashid M., Rashid M.I., Akbar H., Ahmad L., Hassan M.A., Ashraf K., Saeed K., Gharbi M. (2019). A systematic review on modelling approaches for economic losses studies caused by parasites and their associated diseases in cattle. Parasitology.

[23] Hsu Y.W., Tsai C.F., Chen W.K., Huang C.F., Yen C.C. (2011). A subacute toxicity evaluation of green tea (*Camellia sinensis*) extract in mice. Food Chem. Toxicol..

[24] Paveto C., Güida M.C., Esteva M.I., Martino V., Coussio J., Flawiá M.M., Torres H.N. (2004). Anti-*Trypanosoma cruzi* activity of green tea (*Camellia sinensis*) catechins. Antimicrob. Agents Chemother..

[25] Thipubon P., Tipsuwan W., Uthaipibull C., Santitherakul S., Srichairatanakool S. (2015). Anti-malarial effect of 1-(N-acetyl-6-aminohexyl)-3-hydroxy-2-methylpyridin-4-one and green tea extract on erythrocyte-stage *Plasmodium berghei* in mice. Asian Pac. J. Trop. Biomed..

[26] Rodriguez-Vivas R.I., Alonso-Díaz M.A., Rodríguez-Arevalo F., Fragoso-Sanchez H., Santamaria V.M., Rosario-Cruz R. (2006). Prevalence and potential risk factors for organophosphate and pyrethroid resistance in *Boophilus microplus* ticks on cattle ranches from the State of Yucatan, Mexico. Veter-Parasitol..

[27] Vudriko P., Okwee-Acai J., Byaruhanga J., Tayebwa D.S., Okech S.G., Tweyongyere R., Wampande E.M., Okurut A.R.A., Mugabi K., Muhindo J.B. (2018). Chemical tick control practices in southwestern and northwestern Uganda. Ticks Tick Borne Dis..

[28] Freitas D.R., Rosa R.M., Moraes J., Campos E., Logullo C., Da Silva Vaz I., Masuda A. (2007). Relationship between glutathione S-transferase, catalase, oxygen consumption, lipid peroxidation and oxidative stress in eggs and larvae of *Boophilus microplus* (Acarina: Ixodidae). Comp. Biochem. Physiol. Part A Mol. Integr. Physiol..

[29] Hernandez E.P., Kusakisako K., Talactac M.R., Galay R.L., Hatta T., Fujisaki K., Tsuji N., Tanaka T. (2018). Glutathione S-transferases play a role in the detoxification of flumethrin and chlorpyrifos in *Haemaphysalis longicornis*. Parasites Vectors.

[30] Malak N., Niaz S., Wadood A., Nasreen N., Ali I., Iqbal J., Swelum A.A., Ezzat Ahmed A., Alkahtani M.A., Zając Z. (2022). In silico approaches to develop herbal acaricides against *R*. (*Boophilus*) *microplus* and In vitro Anti-Tick activities of selected medicinal plants. Saudi J. Biol. Sci..

[31] Walker A.R., Bouattour A., Camicas J.-L., Estrada-Peña A., Horak I.G., Latif A.A., Pegram R.G., Preston P.M. (2023). Ticks of Domestic Animals in Africa: A Guide to Identification of Species. Proceedings of the Bioscience Reports.

[32] Albus U. (2012). Guide for the Care and Use of Laboratory Animals.

[33] Tamfu A.N., Ceylan O., Kucukaydin S., Duru M.E. (2020). HPLC-DAD phenolic profiles, antibiofilm, anti-quorum sensing and enzyme inhibitory potentials of *Camellia sinensis* (L.) O. Kuntze and *Curcuma longa* L. LWT.

[34] Rubab S., Rizwani G.H., HASSAN M.M.U., Durrani A.I., Hanif U., Ajaib M., Liaqat I., Sadiqa A., Shafi A., BATOOL10 F. (2022). Establishment of pharmacognostic standards of different morphological parts of *Camellia sinensis* L. grown in Pakistan. Pak. J. Bot..

[35] Shah S.B., Parveen Z., Bilal M., Sartaj L., Bibi S., Nasir A., Mahmood A. (2018). Assessment of antimicrobial, antioxidant and cytotoxicity properties of *Camellia sinensis* L. Pak. J. Pharm. Sci..

[36] Anand J., Upadhyaya B., Rawat P., Rai N. (2015). Biochemical characterization and pharmacognostic evaluation of purified catechins in green tea (*Camellia sinensis*) cultivars of India. 3 Biotech.

[37] Arnold K., Bordoli L., Kopp J., Schwede T. (2006). The SWISS-MODEL workspace: A web-based environment for protein structure homology modelling. Bioinformatics.

[38] Berman H.M., Battistuz T., Bhat T.N., Bluhm W.F., Bourne P.E., Burkhardt K., Feng Z., Gilliland G.L., Iype L., Jain S. (2002). The Protein Data Bank. Acta Crystallogr. Sect. D.

[39] Tian W., Chen C., Lei X., Zhao J., Liang J. (2018). CASTp 3.0: Computed atlas of surface topography of proteins. Nucleic Acids Res..

[40] Trott O., Olson A.J. (2010). AutoDock Vina: Improving the speed and accuracy of docking with a new scoring function, efficient optimization, and multithreading. J. Comput. Chem..

[41] Barreira S., Moutinho C., Silva A.M.N., Neves J., Seo E.-J., Hegazy M.-E.F., Efferth T., Gomes L.R. (2021). Phytochemical characterization and biological activities of green tea (*Camellia sinensis*) produced in the Azores, Portugal. Phytomedicine Plus.

[42] Childers M.C., Daggett V. (2017). Insights from molecular dynamics simulations for computational protein design. Mol. Syst. Des. Eng..

[43] López-Blanco J.R., Aliaga J.I., Quintana-Ortí E.S., Chacón P. (2014). iMODS: Internal coordinates normal mode analysis server. Nucleic Acids Res..

[44] El Khetabi A., Lahlali R., Ezrari S., Radouane N., Lyousfi N., Banani H., Askarne L., Tahiri A., El Ghadraoui L., Belmalha S. (2022). Role of plant extracts and essential oils in fighting against postharvest fruit pathogens and extending fruit shelf life: A review. Trends Food Sci. Technol..

[45] Rakshit A., Meena V.S., Abhilash P.C., Sarma B.K., Singh H.B., Fraceto L., Parihar M., Kumar A. (2021). Biopesticides: Volume 2: Advances in Bio-Inoculants.

[46] Kumar J., Ramlal A., Mallick D., Mishra V. (2021). An Overview of Some Biopesticides and Their Importance in Plant Protection for Commercial Acceptance. Plants.

[47] Ayub S., Malak N., Cossío-Bayúgar R., Nasreen N., Khan A., Niaz S., Khan A., Alanazi A.D., Ben Said M. (2023). In Vitro and In Silico Protocols for the Assessment of Anti-Tick Compounds from *Pinus roxburghii* against *Rhipicephalus* (*Boophilus*) *microplus* Ticks. Animals.

[48] Saman S., Chen C.C., Malak N., Khan A., Nasreen N., Khan A., Niaz S., Rehman G., Rodriguez-Vivas R.I., Cossío-Bayúgar R. (2022). Ethanolic Extracts of *Datura innoxia* Have Promising Acaricidal Activity against *Rhipicephalus microplus* as It Blocks the Glutathione S-Transferase Activity of the Target Tick. Genes.

[49] Vishnoi H., Bodla R.B., Kant R., Bodla R.B. (2018). Green Tea (*Camellia sinensis*) and its antioxidant property: A review. Int. J. Pharm. Sci. Res..

[50] Chan E.W., Soh E.Y., Tie P.P., Law Y.P. (2011). Antioxidant and antibacterial properties of green, black, and herbal teas of *Camellia sinensis*. Pharmacogn. Res..

[51] Naveed M., BiBi J., Kamboh A.A., Suheryani I., Kakar I., Fazlani S.A., FangFang X., Kalhoro S.A., Yunjuan L., Kakar M.U. (2018). Pharmacological values and therapeutic properties of black tea (*Camellia sinensis*): A comprehensive overview. Biomed. Pharmacother..

[52] Alemu S., Bayu Y., Wasihun P., Abdurahman A. (2022). Prevalence, Phytochemical Investigation, and In Vitro Acaricidal Efficacy Evaluation of *Dodonaea angustifolia*, *Eucalyptus globulus*, *Millettia ferruginea*, and *Euphorbia abyssinica* against Sarcoptic Mange of Camel, Babile District, Ethiopia. J. Parasitol. Res..

[53] Gu X., Fang C., Yang G., Xie Y., Nong X., Zhu J., Wang S., Peng X., Yan Q. (2014). Acaricidal properties of an *Ailanthus altissima* bark extract against *Psoroptes cuniculi* and *Sarcoptes scabiei* var. cuniculi in vitro. Exp. Appl. Acarol..

[54] Sherwani S.K., Bokhari T.Z., Sualeh M., Kausar R., Muhammad H., Nangyal H., Sarwar M., Kazmi S. (2013). Anti-arthritic and insecticidal property of crude aqueous *Camellia sinensis* (Green tea) infusion and decoction: Study by two in vitro methods. Global Journal of Pharmacology.

[55] Khan S., Nazir M., Raiz N., Saleem M., Zengin G., Fazal G., Saleem H., Mukhtar M., Tousif M.I., Tareen R.B. (2019). Phytochemical profiling, in vitro biological properties and in silico studies on *Caragana ambigua* stocks (Fabaceae): A comprehensive approach. Ind. Crops Prod..

[56] Soh P.N., Witkowski B., Olagnier D., Nicolau M.L., Garcia-Alvarez M.C., Berry A., Benoit-Vical F. (2009). In vitro and in vivo properties of ellagic acid in malaria treatment. Antimicrob. Agents Chemother..

[57] Abuelsaad A.S., Mohamed I., Allam G., Al-Solumani A.A. (2013). Antimicrobial and immunomodulating activities of hesperidin and ellagic acid against diarrheic *Aeromonas hydrophila* in a murine model. Life Sci..

[58] Park S.W., Kwon M.J., Yoo J.Y., Choi H.J., Ahn Y.J. (2014). Antiviral activity and possible mode of action of ellagic acid identified in *Lagerstroemia speciosa* leaves toward human rhinoviruses. BMC Complement. Altern. Med..

[59] Yang D., Wang T., Long M., Li P. (2020). Quercetin: Its Main Pharmacological Activity and Potential Application in Clinical Medicine. Oxidative Med. Cell. Longev..

[60] Bezerra W.A.d.S., Tavares C.P., Rocha C.Q.d., Vaz Junior I.d.S., Michels P.A.M., Costa Junior L.M., Soares A.M.d.S. (2022). Anonaine from *Annona crassiflora* inhibits glutathione S-transferase and improves cypermethrin activity on *Rhipicephalus* (*Boophilus*) *microplus* (Canestrini, 1887). Exp. Parasitol..

